# Reconstruction of Lateral Mandibular Defects with Soft Tissue Loss: The Role of the Submental Flap

**Published:** 2018-07

**Authors:** Amin Rahpeyma, Saeedeh khajehahmadi

**Affiliations:** 1 *Oral and Maxillofacial Diseases Research Center, Mashhad University of Medical Sciences, Mashhad, Iran*; 2 *Dental Research Center, Mashhad University of Medical Sciences, Mashhad, Iran.*

**Keywords:** Flap, Mandible, Reconstruction

## Abstract

**Introduction:**

Mandibular continuity defects after pathologic resections or traumatic events are difficult cases for reconstruction. Defects involving both hard and soft tissue loss are more challenging, because of problems in soft tissue coverage^.^ The role of the submental flap in this regard is presented.

**Materials and Methods:**

In a retrospective study from the archived files of Ghaem Hospital, Mashhad, Iran between 2007–2016, lateral mandibular defects that were managed with submental flap for soft tissue coverage were selected.

**Results:**

Ten patients had been treated, of whom four cases were due to trauma/gunshot events and six cases were defined as pathologic resection; five patients with malignant lesions and one with benign intraosseous pathology, but with soft tissue invasion. There was one complication overall, concerning orocutaneous fistula formation.

**Conclusion:**

Submental flap is indicated for coverage of the reconstruction plate when the lateral mandible is resected/avulsed with soft tissue loss limited to the oral cavity or due to through and through defects in the lower third of the face.

## Introduction

Mandibular continuity defects after pathologic resections or traumatic events are difficult cases, but occasionally present to reconstructive surgeons. Restoring mandibular continuity, maintaining preoperative occlusion, improving mastication, and preventing face disfigurement are the goals of the mandibular reconstruction (1). Defects involving both hard and soft tissue loss are more challenging, because of the problems in soft tissue coverage (2). Soft tissue loss occurs in trauma avulsion (mainly from gunshot) and pathologic resections.Interosseous tumors with soft tissue penetration and malignant mucosal lesions with bone invasion fall within this group (3,4).

Use of a titanium reconstruction plate to regain mandibular continuity and a soft tissue flap for reconstruction of plate coverage, with or without free bone grafting, are common practice (5). Free osteomuscular flaps are now state-of-the-art for mandibular lateral defects accompanied by soft tissue loss, to restore both form and function. These techniques are more complicated, require specially trained surgeons, and are not recommended in patients with poor general health or advanced cancer (6).

In this article, the experience of the authors with respect to an orthograde submental pedicled myocutaneous flap (a soft tissue flap for coverage of the mandibular reconstruction plate), with or without a free corticocancellous bone graft, is described.

## Materials and Methods

In a retrospective study from the archived files of Ghaem Hospital, Mashhad (Iran) between 2007 and 2016, lateral mandibular defects that were managed with submental flap for soft tissue coverage were selected. A lateral defect was defined as a mandibular defect not crossing the midline and not including the mandibular condyle. Patients were recalled, and the following parameters were assessed: etiology, dental state, size of the mandibular defect (cm), right or left side, follow-up years, bone grafting, and the need for a reconstruction plate. Flap viability and complications such as flap necrosis, sloughing, and dehiscence were evaluated.

*Flap anatomy*

A pedicled myocutaneous submental island flap with elliptical design had been used for intraoral reconstruction. The length of the flap was chosen to be slightly larger than the mandibular bony defect and the width of the flap was selected based on a pinch test and the site dimensions of the recipient. In the pedicled side, the anterior belly of the digastric muscle and mylohyoid muscle were included in the flap thickness, while in the non-pedicled side, the flap was composed of platysma muscle and skin. An orthograde variant with preservation of facial artery, vein, and submental vessels was selected.

## Results

Demographic data concerning the patients in whom a submental flap was used for coverage of the mandibular lateral defect are shown in Table 1. Ten patients had been treated, among whom four cases were due to trauma/gunshot events and six cases were classified as pathologic resection; five patients with malignant lesions and one with benign intraosseous pathology, but with soft tissue invasion. 

**Table 1:** Demographic data concerning the patients in whom a submental flap was used for coverage of the mandibular lateral defect

**Patient**	**Age**	**Sex**	**Etiology**	**Dental state**	**Size of mandibular defect (cm)**	**Right or left**	**Follow-up (years)**	**Bone graft**	**Complication**
1	54	M	Trauma	E	6	L	7	+	--
2	27	M	Gunshot	D	6	R	1	+	--
3	40	M	Trauma	D	3	L	7	+	--
4	52	F	SCC	D	4	L	3	--	--
5	70	F	SCC	E	4	L	5	--	--
6	48	F	CCOC	D	4	L	5	+	--
7	67	M	SCC	E	5	L	3	--	--
8	73	F	SCC	E	6	R	5 (dead)	--	--
9	20	M	Gunshot	D	5	L	9	+	Orocutaneous fistula
10	52	F	COC	D	6	L	3	+	--

Abbreviations: D = Dentate; E: Edentulous; M = Male; F = Female; SCC = Squamous cell carcinoma; CCOC = Clear cell odontogenic carcinoma; COC = Calcifying epithelial odontogenic cyst

**Fig1:**
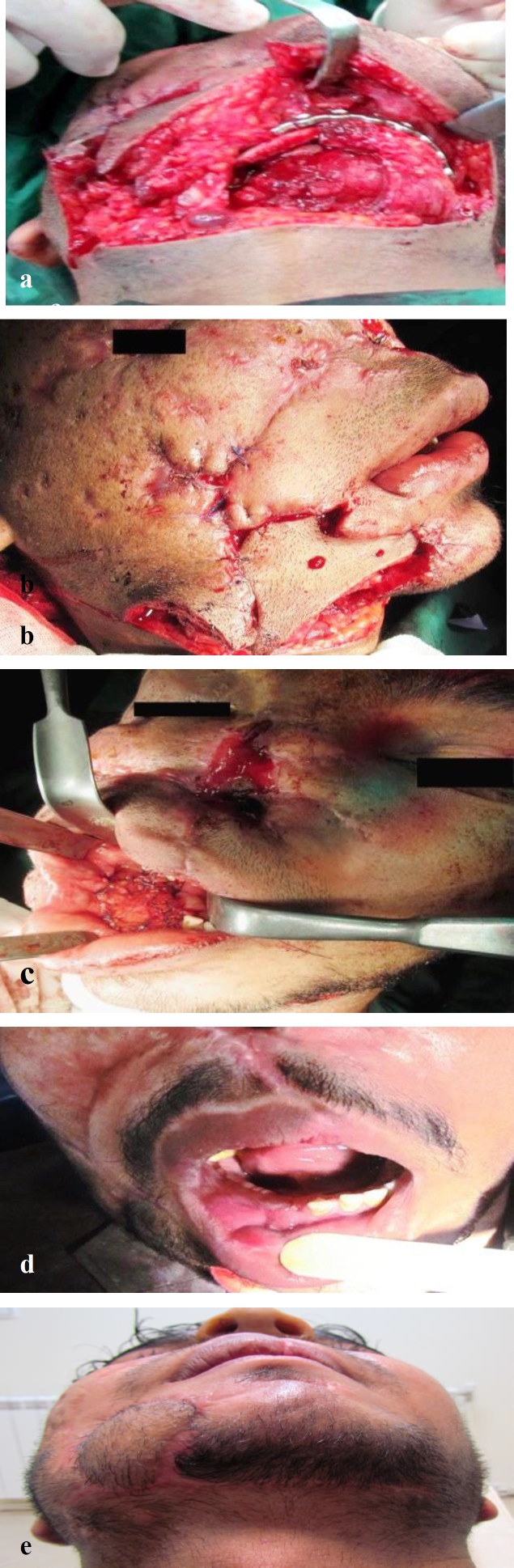
(a) Submental flap is used for coverage of reconstruction plate and free bone graft in a gunshot victim. (b) The hairy part is used for reconstructing through and through skin defect. (c) The deepithelialized part of the flap is used for intraoral reconstruction. (d) Photograph taken 2 months after surgery shows complete epithelialization of the submental flap. (e) Birds-eye view 1 year after surgery shows the hairy part of the submental flap. The avulsed nose is replaced by an implant-supported nasal prosthesis

The gunshot victims had through and through defects. The age range of the patients was 20–73 years. Forty percent of the patients were edentulous, and the male/female ratio was equal. The length of the resected mandible was 3–6 cm. Eighty percent of the submental flaps were used for left side mandibular reconstruction. Follow-up was between 1 and 9 years. A submental flap and reconstruction plate without bone grafting had been used in five patients, and a combination of a free bone graft and reconstruction plate was applied in the other six cases (Fig.1). 

**Fig 2:**
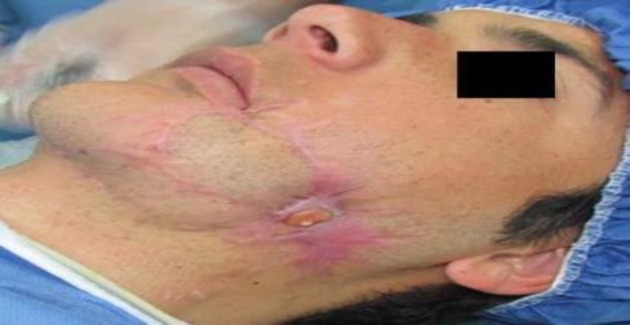
Orocutaneous fistula behind the submental flap

The source of the bone graft was the anterior iliac crest. This flap was used for coverage of the exposed mandibular basal bone in three patients; two after segmental resection and the other after trauma to the mandible. There was one complication; a case of orocutaneous fistula in the composite mandibular gunshot wound with a lateral mandibular defect and associated large-volume cheek skin loss (Fig.2). There were no cases of hematoma or flap loss.

## Discussion

Pedicled myocutaneous flaps such as pectoralis major, deltopectoral, and infrahyoid flap have been indicated for coverage of reconstruction plates used for bridging small and lateral mandibular defects (7–10).

**Fig 3:**
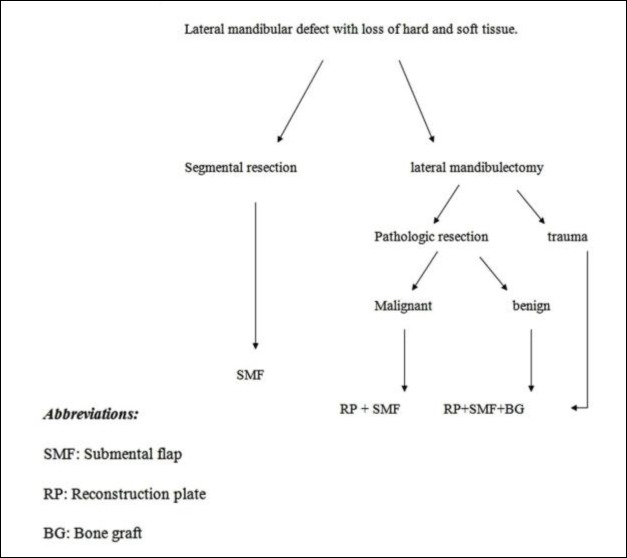
Suggested algorithm for reconstruction of the lateral mandibular defect, accompanied with soft tissue loss by submental flap

Free osteomuscular flaps such as fibula, iliac crest, radial, scapular, serratus anterior and rib, metatarsal, and lateral arm flaps including the humerus were used for mandibular reconstruction, with a failure rate of between 3.4% and 6.2% (11). However, in a comparison between free flaps and submental pedicled flaps, the surgical time and length of hospital stay is reduced in later (12). Submental flap adds a new source of soft tissue in this area, offering benefits of an axial pattern flap with a large paddle size, in the vicinity of the mandible (13,14). The length of the scar in the submentum is slightly longer than when a reconstruction plate is used alone for mandibular reconstruction, so additional scar lines are prohibited. Despite the problem of hairs in male patients, this flap was used for oral cavity reconstruction in males, accounting for half the patients in our series. An algorithm for use of a submental flap in lateral mandibular continuity defects accompanied with soft tissue loss is proposed (Fig.3).

Based on this case study and our wider experience, we are able to make a number of comments about the role of the submental flap in reconstruction of lateral mandibular defects with soft tissue loss. First, in order to recreate alveololingual sulcus, the submental flap should be secured to the holes of the reconstruction plate. Adequate reconstruction of the volume medial to the palate decreases dead space, making intraoral plate exposure less likely. In addition, in patients with malignant oral lesions in which post-operative radiotherapy is in the plane, transfer of the pedicled submental flap from the buccal to the lingual, over the reconstruction plate, is recommended. This maneuver increases the soft tissue thickness, between the reconstruction plate and the outer skin and lessens the risk of extraoral plate exposure after radiotherapy. In male patients with heavy beards, if the submental flap is planned to be used for oral cavity reconstruction, then a deepithelialized variant should be considered to overcome the problem of hairs in the oral cavity (15).

Whenever possible, bone grafting should be considered in treatment planning to restore mandibular continuity and function. Size limitations (below 8 cm) for free bone grafting should be strictly adhered to. Bone grafting is especially important for edentulous patients to help them in using new dentures. This strategy has several advantages including changing the load-bearing nature of the reconstruction plate to a load-sharing one, thus reducing the risk of plate fracture, as well as rendering prosthetic rehabilitation of the mouth possible (16). Free bone grafting is not routine in the management of gunshot avulsions (first stage surgery), and is not the rule. It is advised that bone grafting should be performed even in clinically infected mandibular fractures, if soft tissue coverage is guaranteed (17). 

Finally, we note that tunneling to reach the reconstructed area and reverse flow variant to increase flap mobility may decrease flap perfusion and cause of partial flap necrosis, as reported in literature (16,17). In addition, flap movement in mandibular reconstruction with a submental flap is transposition, and the orthograde variant is the blood supply of this flap. For these two reasons, flap loss did not occur in this study, while securing the submental flap to the reconstruction plate holes prevented hematoma beneath the flap.

## Conclusion

Submental flap is indicated for coverage of the reconstruction plate when the lateral mandible is resected/avulsed with soft tissue loss limited to the oral cavity or through and through defects of the lower third of the face.
